# Fracture Resistance Analysis of CAD/CAM Interim Fixed Prosthodontic Materials: PMMA, Graphene, Acetal Resin and Polysulfone

**DOI:** 10.3390/polym15071761

**Published:** 2023-04-01

**Authors:** Cristian Abad-Coronel, Carolina Calle, Gabriela Abril, César A. Paltán, Jorge I. Fajardo

**Affiliations:** 1 CAD/CAM Materials and Digital Dentistry Research Group, Faculty of Dentistry, Universidad de Cuenca, Cuenca 010107, Ecuador; 2 Faculty of Dentistry, Universidad de Cuenca, Cuenca 010101, Ecuador; 3 New Materials and Transformation Processes Research Group GiMaT, Universidad Politécnica Salesiana, Cuenca 170517, Ecuador; 4 Mechanical Enginnering Faculty, Universidad Politécnica Salesiana, Cuenca 170517, Ecuador

**Keywords:** interim restorations, PMMA, graphene, polysulfphone, acetalic resin, mechanical properties, CAD/CAM materials

## Abstract

The aim of this study was to evaluate and compare the fracture resistance of temporary restorations made of polymethylmethacrylate (PMMA), graphene-modified PMMA (GRA), acetal resin (AR) and polysulfone (PS) obtained by a subtractive technique (milling) using a computer-aided design and manufacturing (CAD/CAM) system of a three-unit fixed dental prosthesis (FDP). Methods: Four groups of ten samples were fabricated for each material. Each specimen was characterized by a compression test on a universal testing machine, all specimens were loaded to fracture and the value in Newtons (N) was recorded by software connected to the testing machine. The fracture mode was evaluated on all samples using a stereomicroscope. Results: There were statistically significant differences (*p* value < 0.005) between PMMA and the other three materials (PMMA: 1302.71 N; GRA: 1990.02 N; RA: 1796.20 N; PS: 2234.97). PMMA presented a significantly lower value than the other materials, and PS showed the highest value. GRA and RA presented a similar range of values but they were still higher than those of PMMA. Conclusions: GRA, RA and PS are presented as valid options within the range of interim milled restorative materials and as alternatives to PMMA.

## 1. Introduction

According to the Glossary of Prosthodontic Terms (GPT), a provisional restoration is a “fixed or removable dental prosthesis that is designed to improve esthetics, stability and/or function for a specific period of time, after which it should be replaced by a permanent dental prosthesis” [1].

The objectives of interim restorations are soft tissue management, protection of the dentin–pulp complex, stability of the tooth in the arch and evaluation of the form and masticatory function of the planned restorations in efficiency and esthetics [2,3,4]. 

Fracture resistance is an important parameter in determining the mechanical strength and stiffness of a material, mainly in the rehabilitation of edentulous sections, prolonged treatment time or in patients with parafunctional habits [5,6,7].

Biocompatibility with the oral environment, as well as integration with hard and soft tissues, are other important characteristics to consider, as certain materials may release components that are potentially toxic to the patient [8]. There are several techniques for the fabrication of provisional restorations, including the indirect technique with the use of CAD/CAM and conventional direct methods [9].

In a digital workflow, obtaining the final product through the CAM process can be subtractive or additive. Within the subtractive process are milling and grinding, methods in which restorations are obtained from a monolithic block or disc of a given material [10]. The conventional method of fabrication involves complex and time-consuming handling procedures with technique-sensitive materials. Therefore, the use of CAD/CAM technology in dentistry in the last decade has become more frequent, overcoming the disadvantages of traditional provisional materials and techniques and facilitating the production method, allowing more efficient treatments with a wide range of materials [11,12].

Polymer-based materials, such as PMMA, are among the CAD/CAM materials of choice for temporary restorations. PMMA CAD/CAM blocks have cross-linked structures, which provide major advantages over conventional polymers. However, their main disadvantages for long-term use are their poor wear resistance, volume shrinkage after polymerization, lack of resistance to fatigue failure and microbial adhesion [13,14]. Currently, there is a remarkable improvement in the variety of materials to be used for temporary restorations and their physicochemical properties. This provides the clinician with a wide range of possibilities to choose from when carrying out restorative treatments. GRA, RA and PS are examples of materials with improved mechanical properties [15,16]. The use of these materials in the fabrication of provisional restorations is not very widespread; therefore, studying them in depth justifies the development of this research.

### 1.1. PMMA

It is a synthetic polymer obtained by free radical addition and polymerization of methyl methacrylate to polymethyl methacrylate. This material is used in medicine, engineering and dentistry. In the latter, it is used in partial denture bases, total prostheses, impression trays, artificial teeth and temporary crowns, among others [9].

Several studies have shown that the properties of PMMA CAD/CAM exceed those of conventionally manufactured PMMA in terms of hardness, flexural strength, impact strength, fracture toughness and durability [8,17,18,19,20]. However, PMMA is a material with reduced mechanical properties, so its use is focused on the manufacture of interim restorations [21].

### 1.2. Graphene

It is a nanomaterial that has carbon atoms among its main structure, with a two-dimensional honeycomb appearance [15,16]. It comes in different forms, such as graphene sheets, graphene oxide (GO) and reduced graphene oxide (rGO). Graphene oxide is a relatively new material, and research on its applications in dentistry is in its early stages [22].

The properties of GO, such as its biocompatibility and biodegradability, Young’s modulus, decreased antimicrobial adhesion, flexibility and transparency, make it a material with potential in prosthodontics. Although it is already used clinically, there is little scientific information, and clinical studies based on the fracture resistance of this material are very limited [23,24,25].

Therefore, it is important to evaluate their physical and chemical characteristics to ensure their safe and reliable use, since it has been shown that adding GO to biomaterials can potentially improve their properties. According to some authors, the incorporation of carbon nanotubes, such as GO, in acrylic resins can improve their mechanical properties and decrease the degree of shrinkage during polymerization [26,27,28,29].

It has been reported that the incorporation of GO in PMMA improves the physical, mechanical, chemical and biological properties of this material. PMMA exhibits better antimicrobial adhesion effects after incorporating GO than those exhibited by PMMA alone, presenting a higher hydrophobicity [30,31].

GRA is a graphene-reinforced biopolymer that has attracted attention due to its aesthetic, mechanical, electrical and thermal properties; additionally, it presents antimicrobial and biocompatibility characteristics [32].

According to the specifications of its commercial manufacturing company, among its uses are single crowns, fixed bridges with more than two pontics, inlays, veneers, complete prostheses and direct and implant-supported rehabilitations [33,34]. However, there is no scientific evidence or studies that prove the statements of the commercial company in terms of its mechanical behavior, which is a fundamental property for any indirect restoration.

### 1.3. Acetal Resin

Also known as polyoxymethylene (POM), it is formed by the polymerization of formaldehyde and is a thermoplastic technopolymer with a monomer-free crystalline structure. POM is a chain of alternating methyl groups linked by an oxygen molecule [34]. It has favorable properties such as high abrasion and impact resistance (69–122 J/m at 23 °C), low modulus of elasticity (2.9 to 3.5 kN/mm^2^), high elastic memory, low thermal conductivity, resistance to organic solvents, oils and water, low toxicity and clinically acceptable color changes after 300 h of thermocycling [35,36].

These characteristics, together with remarkable esthetic performance, make this material a substitute for acrylic resins and metals in many prosthetic applications. Furthermore, being free of monomers, it is a safe alternative for patients allergic to PMMA. It has minimal porosity, so it resists the accumulation of biological materials and, in turn, reduces odor and staining [37,38,39,40].

In the dental field, the most widespread use of this material is in Removable Partial Dentures (RPD). Specifically, it has been used in the manufacture of both retention and support components to improve the esthetics of prostheses [18]. It can also be an alternative to Cr–Co in patients with allergic reactions to this alloy [41]. The use of AR for the fabrication of provisional restorations is not very widespread. The use of this material in the field of removable prostheses is justified by its high resistance to fracture and its chromatic stability.

### 1.4. Polysulfone

It is a high-performance thermoplastic polymer. It is amorphous and has a glass transition temperature of around 186 CO. It has high stiffness and hardness due to its aromaticity and sulfone groups in its composition. This polymer is generally used in biomedical and environmental applications due to its biocompatibility [42,43]. With respect to this characteristic, the requirement for a material to be considered biocompatible is that it must be antibacterial and free of cytotoxicity. These characteristics are important for use in the oral cavity, so PS would be a promising material for the elaboration of temporary prostheses [44,45].

In general, the use of RA, GRA and PS for provisional restorations is not widespread. Although these materials are already used clinically, there is scarce scientific information and even less data demonstrating their mechanical behavior. It is therefore considered important to study their fracture resistance, with the aim of evaluating them as effective alternatives for CAD/CAM interim restorations.

## 2. Materials and Methods

### 2.1. Sample Preparation

Four CAD/CAM polymeric materials for temporary fixed prostheses were selected and are described in Table 1.

### 2.2. Scanning Process, Design and Materialization of Samples

Using a scanner (PrimeScan 2.0 Dentsply-Sirona, New York, NY, USA) a digital impression of the prefabricated model was obtained, prepared with the following protocol: 2 mm of occlusal reduction, 1.5 mm of axial reduction, light chamfered finishing line, parallelism between axial walls of 6 degrees and rounded edges. The model was digitized with design software (InLAB SW 22.0-Dentsply-Sirona, Bensheim, Germany). A three-unit indirect restoration was designed using the biogeneric modality. The design was transferred to an integrated milling unit (MCX5, Dentsply-Sirona, New York, NY, USA) to obtain the samples (*n* = 40).

### 2.3. Fracture Strength Test

A cast metal master die obtained from the initial scan of the original typodont was milled and prepared to support the testing of each interim crown. The temporary fixed crowns supported by the metal die were fixed on the platform of the universal testing machine (Shimadzu AGS-X series Universal Testing Machine; Shimadzu, Tokyo, Japan).

### 2.4. Compression Test

The specimen was subjected to a quasi-static load test at a speed of 0.5 mm/min with a direction parallel to the major axis of the tooth with an initial preload of 10 N using a universal testing machine (Shimadzu AGS-X series Universal Testing Machine; Shimadzu, Tokyo, Japan.) equipped with a 5 kN load cell. The load was applied through a hardened steel pilot punch with a radius of 3 mm applied in the central pit of the crown. The force/displacement of the specimens were determined using the software incorporated in that instrument (Trapezium X Testing Software, Shimadzu, Tokyo, Japan). All specimens were loaded to fracture and the fracture force was recorded in Newtons (N).

### 2.5. Evaluation of the Fracture Mode

The fracture surface of the samples after loading was observed and analyzed using a high-resolution stereomicroscope (Olympus; SZX7, New York, NY, USA).

**Figure 1:**
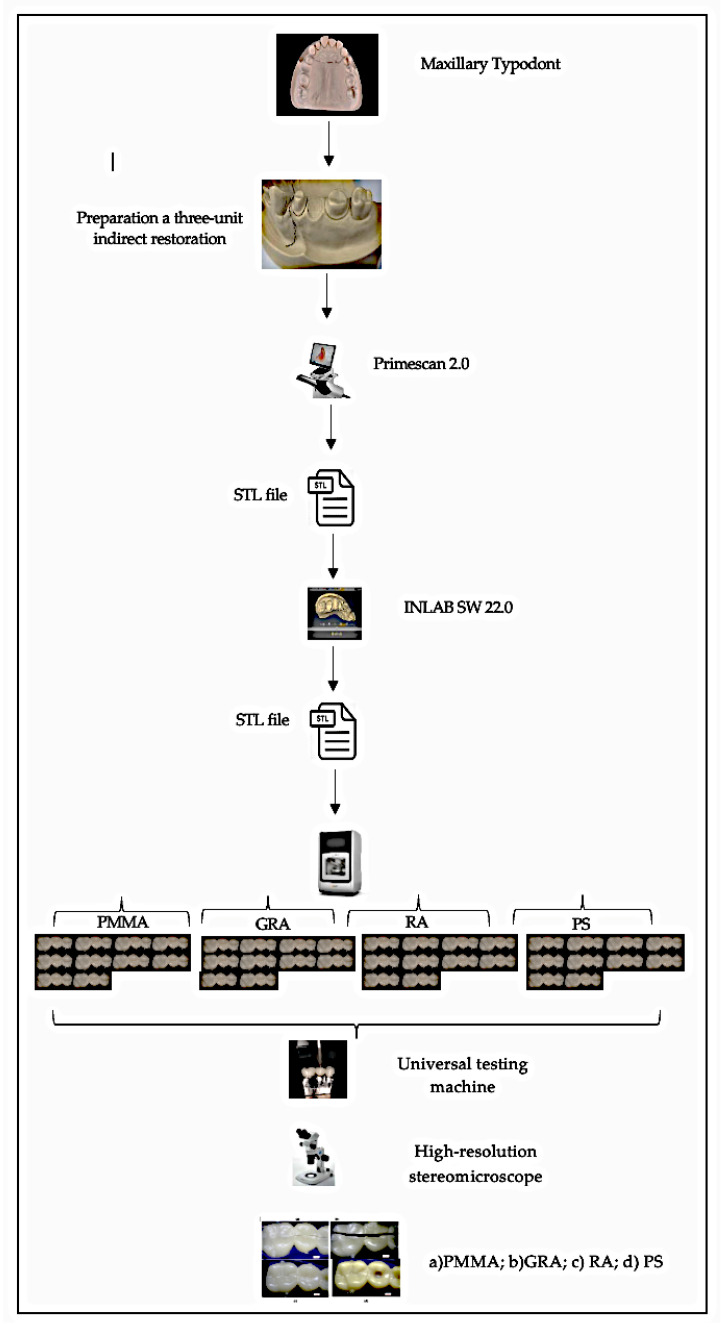
Scheme of the methodology used in this study.

Figure 1 shows an outline of the methodology used for this study.

### 2.6. Data Processing and Statistical Analysis

The data were collected in a data sheet (EXCEL, Microsoft, New York, NY, USA) for descriptive and inferential statistical analysis. Statistical software (SPSS version 27, New York, NY, USA) was used to process the results. Descriptive statistics were used to evaluate the fracture resistance and deformation of the provisional restorations made in PMMA, GRA, RA and PS by digital technology. To make a comparison between the four materials studied, determining which of them had the best fracture resistance and displacement properties, a non-parametric test was used with the Kruskal–Wallis statistic with a significance level of 5%.

## 3. Results

### 3.1. Descriptive Analysis

Table 2 shows a descriptive analysis of the variables to study the fracture resistance of interim CAD/CAM restorations of the four materials studied.

**Figure 2:**
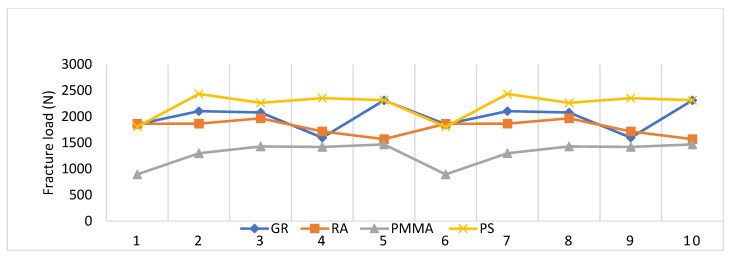
Comparison of fracture strength of GRA, RA, PMMA and PS materials.

Figure 2 shows the distribution of the fracture strength measurements obtained from the different materials, where PMMA showed lower values than the other three materials, which showed similar behavior in the measurements.

**Figure 3:**
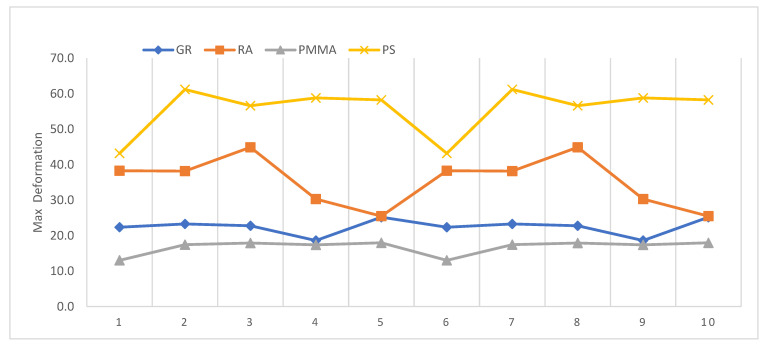
Comparison of the deformation of GR, RA, PMMA and PS materials.

Figure 3 shows the distribution of the maximum deformation measurements obtained from the different materials, where the PMMA material showed lower values than the other three materials and the strain values with the PS material were higher.

### 3.2. Inferential Analysis

The results, considering the strength variable, were significant (*p*-value < 0.005), so the null hypothesis was rejected. The results in Table 3 indicate that there were statistically significant differences between the PMMA material and the other three materials studied.

In Table 3, each row proves the null hypothesis where the distributions of Sample 1 and Sample 2 are equal. Asymptotic significances (bilateral tests) are displayed. The significance level was 0.050. Significance values have been adjusted by Bonferroni correction for various tests.

**Figure 4:**
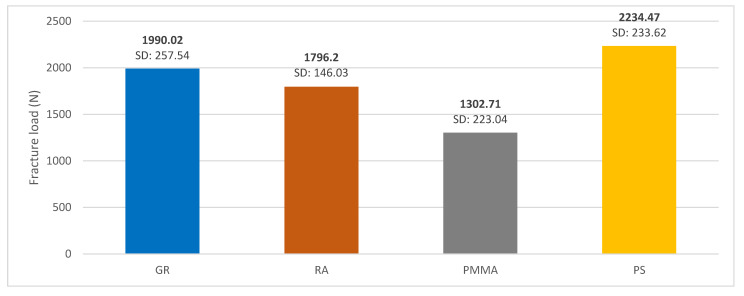
Mean fracture toughness graph of GRA, RA, PMMA and PS materials.

As shown in Figure 4, the material with the highest fracture strength was PS; however, although the GRA and RA materials showed lower fracture toughness, this was not statistically significant (*p*-value < 0.005). The height of the bars shows the average fracture strength for each material.

**Figure 5:**
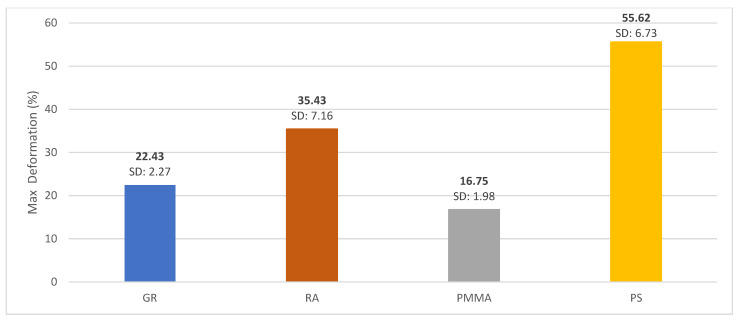
Mean deformation graph for GRA, RA, PMMA and PS materials.

In terms of deformation, PMMA showed less deformation, behaving similarly to GRA. The material with the highest percentage of deformation was PS behaving similarly to RA as shown in Figure 5.

**Figure 6:**
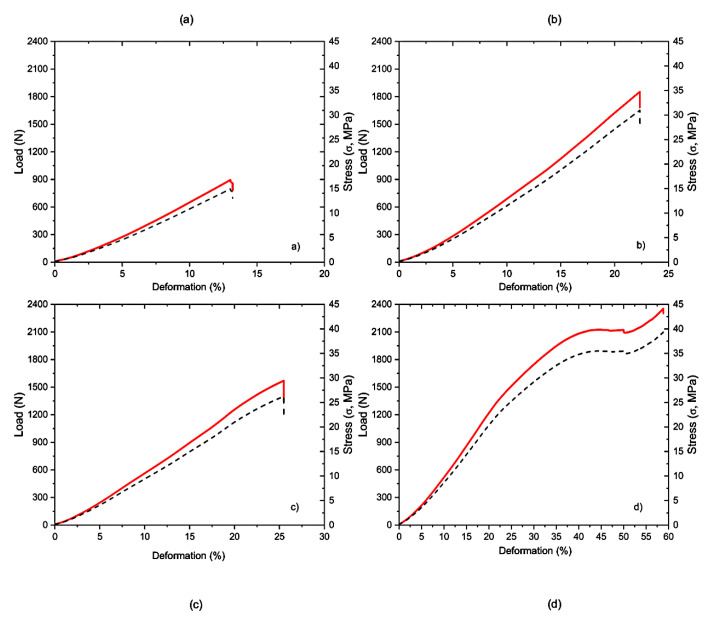
Force-displacement curves of the different materials analyzed: (**a**) PMMA; (**b**) GRA; (**c**) RA; (**d**) PS.

The response of each material studied to the applied load and a comparison between them is shown in Figure 6. We can see that PMMA, GRA and RA have brittle behavior, where RA is the material that presents the highest percentage of deformation. In addition, the material that needs the greatest force to break is GRA. These three materials, having an instantaneous fracture at the moment of reaching the maximum breaking force, are considered brittle, whereas PS is not. Figure 6d shows that after reaching the elastic limit value, the material does not reach fracture; it continues with a crushing within the tested geometry.

**Figure 7:**
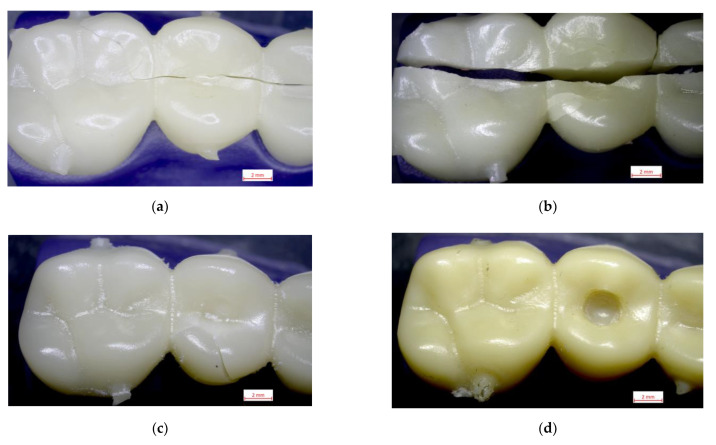
Images of the fracture surfaces of the different materials studied: (**a**) PMMA; (**b**) GRA; (**c**) RA and (**d**) PS.

Figure 7 shows the different fracture behaviors. The most catastrophic and linear failure occurred in GRA, while PS could not fracture despite the applied load, showing a perforation in the sample as shown in Figure 7d. PMMA and RA showed less damage on the fractured surface.

## 4. Discussion

Provisionalization is an important phase in fixed prosthetic treatment protocol. The biological, mechanical and esthetic principles of provisional materials should be considered for the success of restorative treatment [46,47,48].

The literature reviewed indicates that provisional restorations fail more frequently than expected, and the most frequent cause of failure of these restorations is “fracture”, causing patient discomfort and economic loss [47,49,50]. Therefore, it is considered important to investigate and apply advances from dental materials science to the design and construction of interim restorations that offer greater longevity due to their improved biological, mechanical and esthetic properties.

For the present investigation, the fracture strength of four CAD/CAM polymeric materials, PMMA, GRA, RA and PS, was determined and compared in an in vitro study using a three-piece temporary crown bridge. The null hypothesis that the fracture strength of the four materials would not differ was rejected. This is the first attempt in the literature to investigate the performance of RA and PS as potential materials for provisional restorations in the CAD/CAM workflow.

According to the scientific evidence that we have reviewed, the mechanical and physical properties of temporary restorations and fixed prosthetic materials are affected by the fabrication technique and the composition of the materials tested. In several studies, CAD/CAM provisional restorations show higher fracture strength values than restorations fabricated using the direct technique [5,47,51,52]. It is important to mention that with the digital workflow it is possible to obtain standardized material samples with identical thicknesses and sizes, which is considered important to evaluate the mechanical behavior of the materials studied.

PMMA CAD/CAM has been positioned as one of the most common materials for interim prosthesis for long-term use. Several authors mention high fracture resistance values, due to its homogeneous and highly cross-linked structure, and a polymerization process performed under optimized conditions of high pressure and temperature [42,53,54]. However, some authors report certain limitations of the material such as discoloration, hydrolytic degradation and low fracture resistance. Therefore, several studies are currently focused on improving its physical, mechanical and biological properties by incorporating nanoparticles into its structure, such as graphene oxide, to expand its use [25,55,56,57].

In this investigation, PMMA provisional restorations showed lower fracture strength compared to the other three materials studied (1302.71 N) with statistically significant differences (*p*-value < 0.005). These results are close to those reported by Karaokutan, who reported a fracture strength value of 1106 ± 134.65 N for interim crowns fabricated from PMMA CAD/CAM [49]. Other authors mention a mean fracture strength of milled PMMA temporary restorations of 1663.57 N [10]. These variations in fracture strength values could be related to the difference in chemical composition with direct impact on this property. As for the deformation values, the PMMA material showed the lowest deformation (16.75%), behaving similarly to GRA (22.43%). These results differ from those reported in the literature where it is mentioned that the incorporation of GRA nanoparticles improves the dimensional stability of the polymers, which allows the restoration to maintain its shape over time [29].

Graphene oxide is a relatively new material and research into its applications is in its early stages. The potential of GRA to combine with various biomaterials and biomolecules makes it a promising candidate for its enhanced properties, such as mechanical strength, electrical conductivity, thermal stability and biocompatibility [24,58,59,60,61].

There are very few in vitro studies that have analyzed the behavior of GRA combined with other polymers, so the present research allows us to know the mechanical properties of this new material, as an alternative to other more commonly used materials, such as PMMA [57,62].

Some of the most significant properties of GRA are its light weight (one square meter sheet weighs 0.77 milligrams) and its high electrical and thermal conductivity. It is approximately 200 times harder than steel and, consequently, it is much more resistant to wear, compression and tension (120 GPa before breaking) [24]. All this is evidenced in the present investigation, with elevated values in fracture strength with an average of 1990.02 ± 257.54 N being superior to PMMA (1302.71 N), with statistically significant differences (*p* value < 0.005). This is in agreement with that reported by Di Carlo et al., who carried out an in vitro study on twenty rectangular specimens manufactured by a milling machine and divided into two groups (*n* = 10/group): Group 1, PMMA; Group 2, GRA-PMMA. The specimens were subjected to a bending test to evaluate the fracture strength of the materials. The authors reported that each GRA and PMMA specimen showed significantly higher fracture toughness values compared to the PMMA specimens. The bonding between the nano-reinforcement and the polymeric matrix is one of the critical aspects that explains the increased mechanical properties in this type of material [56]. These results suggest that graphene-reinforced PMMA is a promising material to be used for prosthetic purposes. This was demonstrated by a significant increase in both peak load and fracture toughness, which was obtained in the present study as a result of the compression test performed on the GRA-modified PMMA samples. In addition, the latter presented greater homogeneity in their mechanical behavior, which supports the potential value of this material in dental prostheses.

According to some authors, although chemical and mechanical reinforcements with complementary materials have shown remarkable improvements in the mechanical properties of PMMA, it is a challenge to not affect other properties such as color, translucency or biocompatibility. This may be one of the reasons why PMMA remains the material of choice for long-term temporization [42].

Although AR has been studied mainly in the field of RPD, as an alternative to cobalt–chromium [41], there are no studies that analyze AR as a material for provisional CAD/CAM restorations. Therefore, it is difficult to compare the fracture resistance value obtained in the present study with other authors. However, it can be affirmed that, even obtaining a lower value (1796.20 N) compared to GRA (1990.02 N), the differences were not statistically significant (*p* value > 0.005). In comparison with PMMA, RA presented a higher fracture load value with statistically significant differences (*p* value < 0.005). Therefore, this material can be considered as another alternative to PMMA. These results coincide with studies which highlight that the use of this material in the field of fixed prosthesis is justified by the high resistance to fracture and the chromatic stability it presents. This presents this material as an alternative in the elaboration of long-lasting temporary restorations.

In the case of PS, this material presented a ductile behavior (large plastic deformations prior to failure) without showing a fracture phenomenon, but presenting a typical behavior of a compression test with a maximum fracture force of 2434.29 N. Compared with the other three materials studied in this research, PS was the material with the highest percentage of deformation (55.62%), behaving similarly to RA (35.43%). In a study carried out at the University of Sydney in 1984, it was found that PS resists up to four times more than PMMA as a denture base [63]. These results agree with those obtained in the present investigation, in which statistically superior values of resistance to fracture were obtained for PS (2234.47 N) compared with PMMA, which obtained a value of 1302.71 N.

In 2004, Kemp determined that placing PS reinforcement fibers in the PMMA polymeric matrix would improve the flexural strength and flexural modulus of PMMA [64]. It is important to note that although PS responded with adequate mechanical properties in the study, it does not necessarily mean that it is the best material for interim fixed prostheses. It is recommended that practitioners make their selection based on the clinical needs of each situation, physical properties, patient response, appearance of the material, durability of the restoration and cost, among others.

An important aspect to consider is that the average values of occlusal forces reported in the literature are 350 N in the molar area and 250 N in the incisor area. However, patients with bruxism may have much higher forces and, in the literature, these values are reported to increase to 720–900 N [65,66,67]. Considering that the fracture values exceeded the maximum masticatory forces in the posterior region of about 900 N, all the materials studied have the potential to resist the forces that occur clinically.

This study, as with all in vitro research and without a simulation of the oral environment, presented limitations. In addition, it should be mentioned that for the compression test, a maximum load was used that did not manage to catastrophically fracture the PS material; in subsequent research, another type of force could be used to evaluate and compare it with other materials. It is also important to mention that future work should investigate other mechanical properties of these materials such as fatigue, wear resistance, microhardness and hardness for a more complex analysis of milled CAD/CAM temporary materials.

## 5. Conclusions

1. PMMA interim restorations materialized using a subtractive technique (milling) using a CAD/CAM system showed the lowest load to fracture compared to GRA, RA and PS.
2. Provisional restorations of GRA and RA materialized by subtractive technique (milling) using a CAD/CAM system showed similar load-to-fracture and deformation behavior exceeding the values obtained with PMMA.
3. The interim restorations of PS materialized by subtractive technique (milling) using a CAD/CAM system showed a very ductile behavior without reaching fracture, presenting the highest percentage of deformation.
4. GRA, RA and PS are presented as valid options within the range of restorative materials and as alternatives to PMMA. However, further studies are needed to evaluate their resistance over time and their clinical use.

## Figures and Tables

**Table 1:** Summary of materials used in the study.

Product Name	Model	Batch	Color
PMMA	2112-A2-18	2021/31	A2
GRA	GCAM	22041120101	A1
RA	G5008	2/0141/00	A1
PS	GT MEDICAL PS	N/A	A3

**Table 2:** Descriptive summary of the fracture resistance and deformation of the four materials studied.

Group	Statistics	Strength (N)	Max-Deformation (%)
GRA	Mean	1990.02	22.43
	Standard deviation	257.54	2.27
	VC *	12.90%	10.10%
	Minimum	1597.58	18.61
	Maximum	2312.32	25.2
	Confidence Interval 95%	(1805.8; 2174.3)	(20.8; 24.1)
RA	Mean	1796.2	35.43
	Standard deviation	146.03	7.16
	VC	8.10%	20.20%
	Minimum	1569.81	25.47
	Maximun	1967.42	44.9
	Confidence Interval 95%	(1691.7; 1900.7)	(30.3; 40.5)
PMMA	Mean	1302.71	16.75
	Standard deviation	223.04	1.98
	VC	17.10%	11.80%
	Minimum	894.91	13.02
	Maximun	1468.43	17.98
	Confidence Interval 95%	(1143.1; 1462.3)	(15.3; 18.2)
PS	Mean	2234.47	55.62
	Standard deviation	233.62	6.73
	VC	10.50%	12.10%
	Minimum	1805.21	43.17
	Maximum	2434.29	61.2
	Confidence Interval 95%	(2067.3; 2401.6)	(50.8; 60.4)

* VC: Variation coefficient.

**Table 3:** Paired comparison test summary.

Group1-Group 2	Statistic Test	Statistic Test SD	Sig.
PMMA-RA	14,000 *	2680	0.007
PMMA-GR	19,200 *	3676	0.000
PMMA-PS	−26,800 *	−5131	0.000
RA-GR	5200	996	0.319
RA-PS	−12,800	−2451	0.014
GR-PS	−7600	−1455	0.146

* They showed statistically significant differences..

## Data Availability

Data are contained in the article.
